# Heidelberg Consensus Recommendations on Trustmarks

**DOI:** 10.2196/jmir.2.suppl2.e12

**Published:** 2000-09-13

*In a workshop discussing the worldwide first publicly funded international trustmark project for health information on the web (MedCERTAIN), workshop participants discussed aims, possible beenfits and concerns relating to trustmarks for health information on the web. The following document attempts to summarize the issues raised by participants.*

## Definition of a trustmark

Any certificate, symbol, sign, based on information or meta-information (information about information) provided by a third-party that is aiming

- to enhance peoples trust into a certain product, service, relationship, information provider or piece of information perceived as being trustworthy by the organisation issuing the trustmark; and/or
- to enhance the ability of people to evaluate the trustworthiness of information, services or products.

## Purpose of a trustmark for health information on the web

There is agreement that in medicine there is no absolute truth, so that in practice the evaluation of quality criteria such as "accuracy" of health information poses huge practical barriers. In addition, information on the web is dynamic in nature. The primary aim of a trustmark would therefore not be to provide a guarantee for information correctness or usefulness, but to enhance trust into the information provider.

## Potential benefits of trustmarks

As has been stated in the Heidelberg Collaboration statement of purpose, we agree that an important goal for us is to assure and to continuously improve the quality of health information on the Internet.

We agree that there could be a role for a trustmark in this process,

- as health information providers may learn from the interaction between the experts from the organisation issuing the trustmark, the information provider and the user
- as the mere existence of a trustmark may be an additional incentive for a health information provider to improve internal quality management procedures and to adhere to principles of best practice
- as a trustmark may contribute to protect people and enhance their health by providing information for decision making

## Problems

**1. Lack of experience with trustmarks and lack of evidence for the effectiveness or impact of trustmarks**

We also agree that we have no actual evidence for saying whether and under which conditions trustmarks may create more benefit than harm, and that any project trying to implement a trustmark concept has to be carefully evaluated for the impact of its service on people and information providers.

**2. Business models**

Another problem is the absence of experience with business models which could make sure the financial viability and internal quality control of such services.

A business model that is based on letting information providers pay for evaluation should not disadvantage information providers which are unable to pay a fee.

**3. Legal problems**

Any trustmark concept should take into account potential legal risks, possible risk management strategies and, where appropriate, dispute resolution mechanisms.

*Participants:*

Lucas M. Bachmann (Horten Zentrum für praxisorientierte Forschung und Wissenstransfer, Switzerland), Carl R. Blesius (MedCERTAIN, Germany), Markus Blume (mymedia GmbH, Germany), Carl J. Brandt (NetDoktor.com, Denmark), Dan Brickley (MedCERTAIN, United Kingdom), Alejandro Cacherosky (Buenos Aires Health Secretariat, Argentina), Ken Campell (Leukaemia Research Fund, United Kingdom), Richard Cleland (Federal Trade Comission, U.S.A.), Phil Cross (MedCERTAIN, United Kingdom), Elenice de Castro (PAHO/WHO Pan American Health Organization / World Health Organization, Brazil), Guy de Roy (Ordre des Medecins Conseil National, Belgium), Tony Delamothe (BMJ, United Kingdom), Martin D. Denz (Swiss Medical Informatics Association / Universitätsspital Zürich, Switzerland), Persephone Doupi (Erasmus University, Netherlands), Joan Dzenowagis (WHO World Health Organization, Switzerland), Christer Edling (The Swedish Society of Medicine, Sweden), Gunther Eysenbach (University of Heidelberg / MedCERTAIN, Germany), Gerard Freriks (TNO, Netherlands), Franz Frühwald (Internationales Büro der Österreichischen Ärztekammer, Austria), Lisa Gray (BIOME, United Kingdom), Pelle Gustafsson (Swedish Medical Association, Sweden), Gerhard Heine (European Commission, Luxembourg), Katrin Hörner (Arztpartner / Almeda, Germany), Robert Hsiung (University of Chicago, U.S.A.), Jostein Ingulfsen (Norwegian Board of Health, Norway), Thomas Isenberg (German Association of Consumer Organisations - Arbeitsgemeinschaft der Verbraucherverbände, Germany), Edward Jacob (KNMG, Netherlands), Alex R. Jadad (Canadian Cochrane Centre, Canada), Jacobo Kelber (ensalud.net, Mexico), Hugo Kitzinger (mymedia GmbH, Germany), Inge Kokot (Deutsches Grünes Kreuz e.V., Germany), Hans-Joachim Koubenec (Stiftung Warentest, Germany), Michel Labrecque (Université Laval Québec, Canada, Canada), Kristian Lampe (Finnish Office for Health Technology Assessment / MedCERTAIN, Finland), Stephane Lejeune (Swiss Cancer League, Switzerland), Leonard B. Lerer (INSEAD, France), Odile Leroy (PasteurMed, France), Nicolas Lienert (Medgate AG, Switzerland), Pål Lindström (LocusMedicus, Sweden), Sándor Lipp (Hungarian Ministry of Health, Hungary), Leena Lodenius (Finnish Duodecim Medical Society, Finland), Antti Malmivaara (Finnish Institute of Occupational Health, Finland), Miquel Angela Mayer Pujadas (Official Medical College of Barcelona, Spain), Peter Mills (EncycloMedica Ltd, United Kingdom), Cesar Molinero (Planet Medica, Belgium), Marc Muret (Zürcher Aerzte für Klassische Homöopathie, Switzerland), Wolfgang Nagel (GesundheitScout24 GmbH, Germany), Tim Nater (Health on the Net Foundation HON, Switzerland), Joerg Nitzsche (Zentralbibiliothek für Medizin Koeln, Germany), Debra O´Connor (La Trobe University, Australia), Gert Purkert (Arztpartner / Almeda, Germany), Ramon Sarrias Ramis (Official Medical College of Barcelona, Spain), Christine Reuter (A MedWorld AG, Germany), Ahmad Risk (Internet Healthcare Coalition, United Kingdom), Carl Bénédict Roth (GetWellness, Switzerland), Sebastian Schmid (Arztpartner / Almeda, Germany), Christiane Schmitz (Heinrich Heine Universität, Germany), Luk Schoonbaert (Belgacom Multimedia Ventures, Belgium), Frank Schuler (gfp Kommunikation Köln, Germany), Ulrich Schwanke (DocCheck, Germany), Sasha Shepperd (Imperial College School of Medicine, United Kingdom), Myra Sidrassi (MedCERTAIN, Germany), Chris Sigouin (McMaster University, Canada), Chris Silagy (Monash Medical Centre, Australia), Denise Silber (Internet Healthcare Coalition, U.S.A), Martin Sonderegger (Universitätsspital Zürich, Switzerland), Anke Steckelberg (Universität Hamburg, Germany), Frederik Tautz (Heinrich Heine Universität, Germany), Nicolas P. Terry (Center for Health Law Studies, St. Louis University, U.S.A.), Christian Thomeczek (Ärztliche Zentralstelle Qualitätssicherung, Germany), Wouter Tukker, (Praha Communications, Czech Republic), Gerard H. van der Zanden (The Netherlands Institute for Health Promotion and Disease Prevention NIGZ, Netherlands), Georg von Below (Swiss Medical Assoc./FMH, Switzerland), Frank von Danwitz (Deutsches Diabetes-Forschungsinstitut, Germany), C.-Peter Waegemann (Medical Records Institute, U.S.A.), Thomas Wetter (Dept. Of Medical Informatics, Universität Heidelberg, Germany), Petra Wilson, (European Commission, Belgium), Margaret A. Winker (JAMA, U.S.A.), Jeremy Wyatt (University College London, United Kingdom), Gabriel Yihune (MedCERTAIN, Germany)

